# The Case for Using the Repeatability Coefficient When Calculating Test–Retest Reliability

**DOI:** 10.1371/journal.pone.0073990

**Published:** 2013-09-09

**Authors:** Sharmila Vaz, Torbjörn Falkmer, Anne Elizabeth Passmore, Richard Parsons, Pantelis Andreou

**Affiliations:** 1 School of Occupational Therapy and Social Work, Centre for Research into Disability and Society, Curtin University, Perth, Western Australia, Australia; 2 School of Occupational Therapy and Social Work, Curtin Health Innovation Research Institute, Curtin University, Perth, Western Australia, Australia; 3 School of Occupational Therapy, La Trobe University, Melbourne, Vic. Australia; 4 Rehabilitation Medicine, Department of Medicine and Health Sciences (IMH), Faculty of Health Sciences, Linköping University & Pain and Rehabilitation Centre, UHL, County Council, Linköping, Sweden; 5 Department of Community Health and Epidemiology, Dalhousie University, Halifax, Nova Scotia, Canada; RAND Corporation, United States of America

## Abstract

The use of standardised tools is an essential component of evidence-based practice. Reliance on standardised tools places demands on clinicians to understand their properties, strengths, and weaknesses, in order to interpret results and make clinical decisions. This paper makes a case for clinicians to consider measurement error (ME) indices Coefficient of Repeatability (CR) or the Smallest Real Difference (SRD) over relative reliability coefficients like the Pearson’s (r) and the Intraclass Correlation Coefficient (ICC), while selecting tools to measure change and inferring change as true. The authors present statistical methods that are part of the current approach to evaluate test–retest reliability of assessment tools and outcome measurements. Selected examples from a previous test–retest study are used to elucidate the added advantages of knowledge of the ME of an assessment tool in clinical decision making. The CR is computed in the same units as the assessment tool and sets the boundary of the minimal detectable true change that can be measured by the tool.

## Introduction

### Reliability and Test–retest reliability

Reliability refers to the reproducibility of measurements [1]. Measurements are considered reliable if they are stable over time in stable subjects, show adequate levels of measurement variability, and are sensitive (precise) enough to detect Minimum Clinically Important Difference (MCID) [2,3]. Test–retest reliability or reproducibility is a method of estimating a tool’s reliability by administering it to the same person or a group of people, in the same way, on two or more different occasions, hours or days apart [1]. Test–retest reliability provides clinicians with assurance that the tool measures the outcome the same way, in a stable client, each time it is used. Better reproducibility suggests better precision of single measurements, which is a requirement for better tracking of changes in measurements in research or practice settings [4]. There are two necessary assumptions in test–retest reliability. The first is that the true score does not change between administrations. The second is that the time period between administration is long enough to prevent learning, carry-over effects, or recall [5]. An understanding of the stability or variability in the outcome being measured, and characteristics of participants involved in the reliability study should guide the time interval between administrations.

Perfect test–retest reliability scores are rare, as all instruments respond with some error. Thus, any observed score (O) can be assumed to have a true score (T) and an error component (E) [O = T ± E] [1]. Since it is impossible to know T; the true reliability of any test is not calculable [6]. Reliability can be defined using the statistical concept of variance. It is expressed as the ratio of the variance of T to the variance of O [1]. If the error component is large, then the ratio (reliability coefficient) is close to zero, but it is close to one if the error is relatively small. T is the measurement of a person’s actual ability or status, while O is the score readings provided by the tool. For example, in the case of functional independence, irrespective of assessment tool used, an assumption is made that the client has a ‘true’ functional independence score which reflects his/her functional abilities when perfectly measured [7]. In theory, the same T would be obtained if a client was assessed an infinite number of times [6]. Clinically, it is neither practical nor possible to take infinite measurements; hence, it is impossible to know if the observed score is in fact T. Practitioners make an assumption that a single observation on a client (O) is an accurate estimate of the client’s T (i.e., O = T).

Test–retest reliability is concerned with the repeatability of observations made on or by individuals. It is assumed that O is an accurate measurement of T. When a standardised tool is used to measure an outcome, clinicians rely on the published test–retest reliability coefficient of the tool to guide the confidence in their results.

### Quantifying Test–Retest Reliability

#### Relative reliability

**Figure 1:**
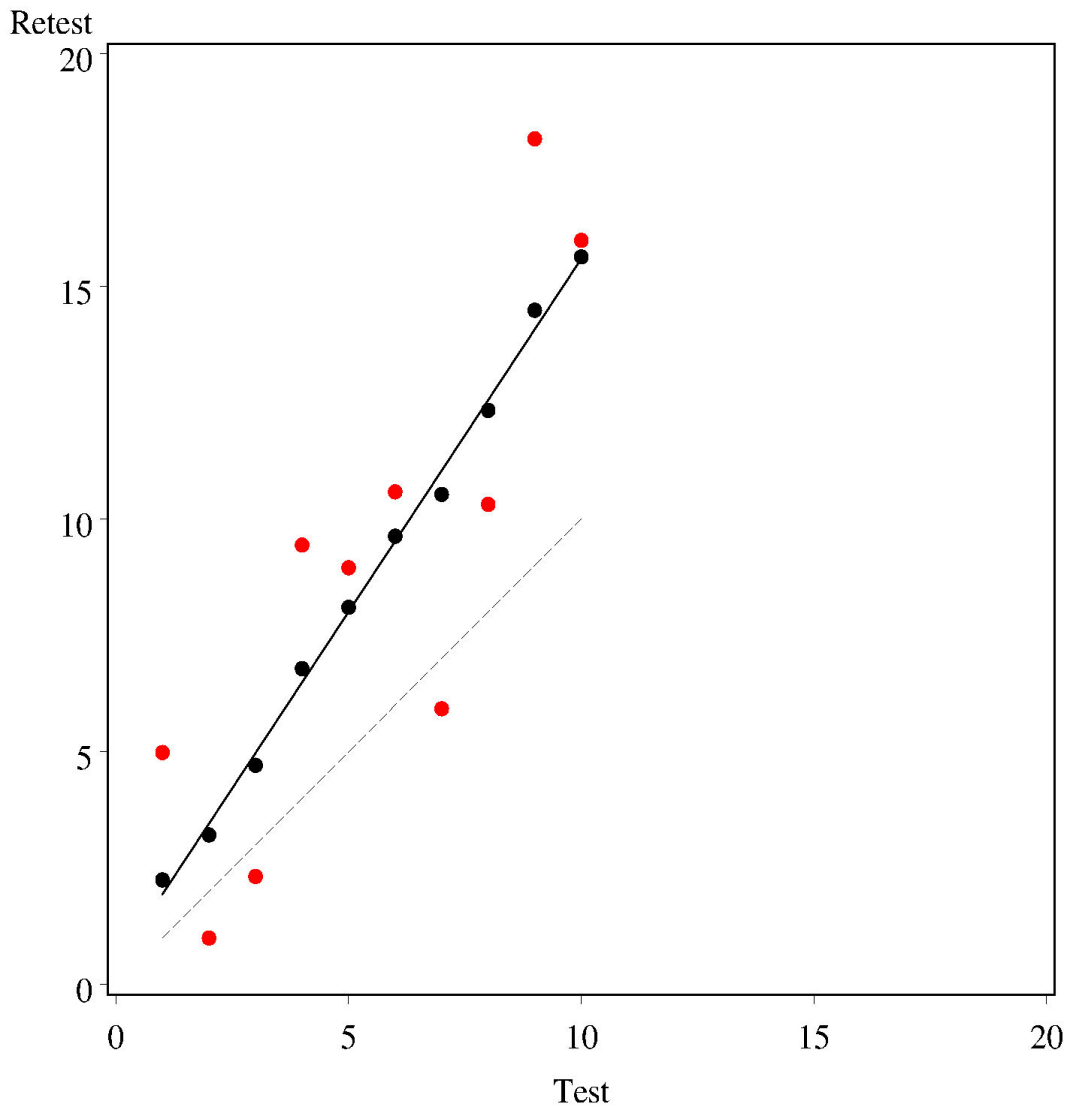
Hypothetical data demonstrating good relative reliability(r = 0.94) due to identical rank ordering of test–retest scores.

Test–retest reliability can be estimated using relative and absolute indices [8]. Relative reliability estimates concern consistency or association of position of individuals in a group, relative to others. Pearson’s Product Moment Correlation coefficient [Pearson’s (*r*)] and the Intraclass correlation coefficient (ICC) are the commonly used relative reliability indices. These correlations quantify the direction (+/-) and the strength of the relationship between test–retest scores by estimating their linear relationship, and lies between +1 and -1 [1]. Perfect correlation is one special case of this, but *r* = +1 is not necessarily an indication of complete agreement (interchangeability) between test–retest scores. The correlation coefficient is a reflection of how closely a set of paired observations (test–retest data in this case) follow a straight line, regardless of the slope of the line. For example, Figure 1 shows two fictional sets of data (black and red circles) which both exhibit a similar linear relationship. The line of best fit is the solid line in the graph and is the same for both datasets, but the black circles sit much closer to the line than the red circles, leading to a much higher correlation coefficient (*r* = 0.99 and 0.84 respectively). Neither sets of circles are on the line of complete agreement (represented by the dashed line in the graph). The difference between correlation and agreement has been eloquently described by Bland & Altman (1999), and Figure 1 is a fictional example to demonstrate this concept. Other authors have remarked on the use of the correlation coefficient as the sole index of test–retest reliability [2,9–11].

The main drawback of Pearson’s (r) value is that it does not provide clinicians with any insight into systematic errors that may be inherent in the measurement obtained with a specific assessment tool. For example, as shown in the hypothetical data set in Figure 1, Pearson’s (r) gives a very high value of 0.99 for the black circles despite the divergence of the measurements from the line of agreement. Clinicians may mistake this excellent correlation for complete agreement between the scores, which is clearly not the case.

Although it is still not a measure of absolute agreement, the Intraclass correlation coefficient (ICC) is often reported in place of the Pearson’s (*r*). The ICC is used frequently to calculate the correlation between more than two sets of measurements (typically in the case of more than two clinicians completing an assessment on a set of individuals) [2,10].

Unlike Pearson’s (*r*), the Intraclass Correlation Coefficient (ICC) accounts for both consistency of performances from test to retest (within-subject change), as well as change in average performance of participants as a group over time (i.e., systematic change in mean) [2,10,12]. There are numerous versions of the ICC, each appropriate to specific research design situations [13]. Both Pearson’s (r) and ICC are influenced by how similar (or different) participants in the research study scored to each other on the outcome being measured (i.e., consistency in the research participants’ scores) [10]. All else being equal, the more similar participants score to each other as a group (i.e., more homogeneous), the smaller the magnitude of Pearson’s (r) and ICC. The magnitude of both Pearson’s (r) and ICC is influenced by outlier scores. When reading reliability studies, and before selecting a tool for use, it is therefore important that practitioners critically review the characteristics of the research participants involved in the reliability estimation study. For example, cognitive function scores of people with advanced Alzheimer’s disease will be more similar to each other than those of a group of people with various neurological conditions at various times since diagnosis. Thus, practitioners need to carefully ensure that the tool selected for use has been tested on a sample group with similar characteristics.

#### Absolute reliability

Absolute reliability is concerned with variability due to random error [8]. Consequently, an absolute reliability index is affected by the degree to which measurements vary, with the premise being the less the variability, the higher the reliability. For example, in the case of goniometry, the margin of error is generally accepted to be 5 degrees for measurement of joints’ Range of Movement (ROM) in the hand, provided the measurements are taken by the same examiner using standardised techniques [14]. This means that while using a goniometer for hand ROM, scores that differ by more than 5 degrees can be considered to reflect a real difference.

**Figure 2:**
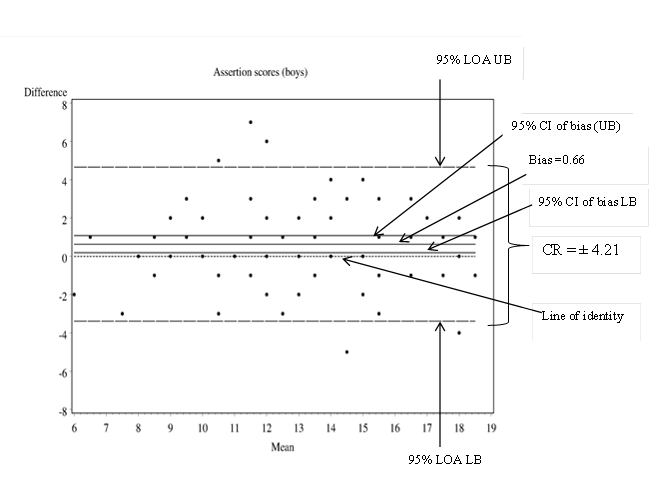
Bland and Altman difference plots using boys’ Times 1 and 2 assertion frequency scores on the SSRS-SSF [15].

**Figure 3:**
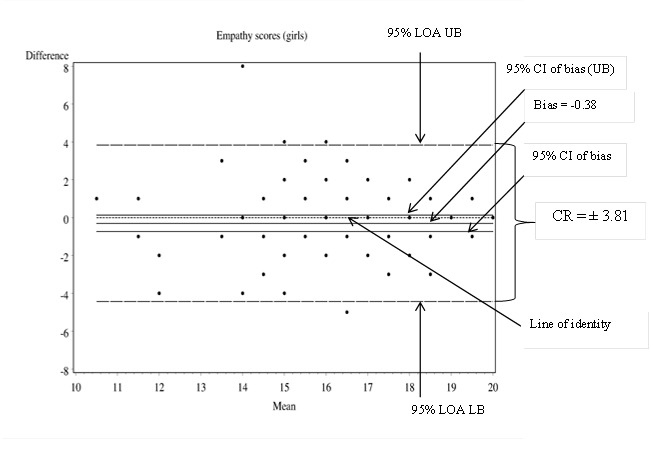
Bland and Altman difference plots using girls’ Times 1 and 2 empathy frequency scores on the SSRS-SSF [15].

The ‘repeatability coefficient’ (CR) also referred to as the Smallest Real Difference (SRD) is a useful index that quantifies absolute reliability ME in the same units as the measurement tool [2,10,11]. The CR of a tool is directly related to the 95% Limits of agreement (LOA) proposed by Bland and Altman (Figures 2 and 3) that contain 95% of differences between repeated measurements on same subjects [2,10,11].

The CR is the value below which the absolute differences between two measurements would lie with 0.95 probability [16,17]. It is calculated by multiplying the within-subject standard deviation (S_W_) or the Standard Error of Measurement (SEM) by 2.77 (√ 2 times 1.96). Thus, CR = 2.77S_w_ [2,11,17]. Both random and systematic errors are taken into account in the CR score [4]. For example, in the case of goniometry, clinicians can be 95% confident that 10 degree change in hand ROM represents 5 degree true change (because we are aware that a goniometer has an established ME of 5 degrees). Because the CR is quantified in the same units as the assessment tool, it lends itself for easy clinical interpretation, and can be used to guide decision making with individual clients on a day to day basis.

To further test the case of the CR over Pearson’s *r* and ICC while selecting outcome tools to measures change and inferring change as true, this paper uses test–retest data from a previous study using the Social Skills Rating System (SSRS-SSF) [15].

## Materials and Methods

A ‘4-week’ test–retest design was used. The secondary level student version of the SSRS-SSF was administered to 187 year 7 students (Mean age = 12 years 3 months, *SD* = 3.93 months) from five randomly selected schools in metropolitan Perth, Western Australia. Detailed information on the present study’s methodology and results has been published elsewhere [15].

This study is based on secondary data analysis of a prior submission entitled Internal consistency, test–retest reliability and measurement error of the self-report version of the Social Skills Rating System in a sample of Australian adolescents [15]. Informed written consent was obtained from school principals, parents and involved students. In situations where the student declined to participate, even with parental consent, they were not included. Students were made aware that they were not obliged to participate in the study, and free to withdraw from this study at any time without justification or prejudice. At all stages, the study conformed to the approved National Health and Medical Research Council Ethics Guidelines [18]. Full ethics approval was obtained from Curtin University Health Research Ethics Committee (Reference number HR 194/2005).

### Data analysis

Analyses were undertaken using SPSS version17 and SAS Version 9.2 software packages. Test–retest reliability estimates, such as the Pearson’s correlation coefficient (*r*), two-way random effects model ICC _(2,1)_ and the CR were computed using standard formulae [2,11,17].

## Results

### Indices of relative reliability

For purposes of illustration, retest estimates from the empathy subscale for girls and assertion subscale for boys are discussed. These subscales were chosen for purposes of graphical emphasis, as participants’ mean scores differed significantly across administrations.

As shown in Table 1, the 4-week ICC of the empathy subscale (for girls) was 0.55 (Pearson’s *r* = 0.55, n = 92) while that of the assertion subscale (for boys) was 0.79 (Pearson’s *r* = 0.80, n = 84). If we refer to the thresholds suggested by Vincent [19] that are typically recommended for individual decision-making in a clinical setting, the social skills scale and subscale test–retest indices were too low to permit reliable use of the SSRS-SSF in practice. However, if we refer to the lower thresholds typically referenced by health professionals [13], the assertion subscale test–retest reliability in boys would be considered to be excellent (*r* = 0.78), while the empathy subscale retest reliability for Year 7 girls would fall into the fair to good category (*r* = 0.54).

**Table 1:** Comparison of measures of reliability for selective social skills Frequency rating scale [15].

**Frequency rating scale**			**Time 1**		**Time 2**			**Relative and absolute reliability indices**										
	**GENDER**	***N***	**Mean**	**SD**	**Mean**	**SD**	**α**	**r**	**ICC _(2,1)_**	**Mean diff (Bias)**	**SD_diff_ between subject**	**t**	**p-value**	**95%LOA (95% CI) LB**	**95%LOA (95% CI) UB**	**Within –subject Variance (WSV)**	**SEM= √(WSV)**	**CR**
**Assertion subscale**	M	84	13.24	3.11	13.90	3.10	0.89	0.78	0.77	0.66	17.20	2.90	0.005	-3.4 (-4.1 to -2.6)	4.7 (3.9 to 5.5)	2.30	±1.52	±4.21
	F	74	12.86	3.07	13.27	3.07	0.84	0.72	0.72	0.40	16.22	1.52	0.13	-4.1 (-5.0 to- 3.2)	4.9 (4.0 to 5.8)	2.66	±1.63	±4.52
**Empathy subscale**	M	98	14.44	2.95	13.95	3.06	0.78	0.62	0.62	-0.49	14.64	-1.86	0.06	-5.6 (-6.5 to -4.7)	4.6 (3.7 to 5.5)	3.49	±1.87	±5.18
	F	92	16.66	1.93	16.27	2.04	0.71	0.54	0.53	-0.38	6.07	-1.89	0.06	-4.1 (-4.8 to -3.4)	3.4 (2.7 to 4.1)	1.89	±1.37	±3.81

ICC_2, 1_ Intraclass correlation coefficient: two-way random effect model (absolute agreement definition)

95% LOA LB (95% CI of the LOA) = Bland and Altman 95% Limits of agreement Lower Boundary (95% Confidence intervals of the limits of agreement)

95% LOA UB (95% CI of the LOA) = Bland and Altman 95% Limits of agreement Upper Boundary (95% Confidence intervals of the limits of agreement)

CR = 2.77 × SEM [15]

### Indices of absolute reliability

As shown in Table 1, as a group, boys had higher assertion scores on retest (Mean bias = 0.66, *p* = .005), despite the expectation that there should be no significant change in assertion scores over 4-weeks (see discussion section) [15]. The reliability coefficient (CR) of the assertion subscale for Year 7 boys was ± 4.21 [2,11]. The observed bias on the assertion subscale (0.66 units) was within the subscale’s ME (± 4.21). In the case of girls, there was no statistical change in mean empathy scores over time (Mean bias = -0.38; *p* = 0.06 and the ME of the empathy subscale was ± 3.81 units.

## Discussion

As presented in Table 1, the 4-week ICC for the empathy subscale (for girls) was 0.53 while that of the assertion subscale (for boys) was 0.77. This means that 53% of variance in the observed empathy scores is attributable to variance in the true score, after adjustment for any real change over time or inconsistency in subject responses over time. The remaining 47% of the observed score variation in either Time 1 or Time 2 represents error, if we assume that no real change would have occurred in the outcome over this short time period. In the above example, based on the magnitude of the ICC of the empathy subscale, a clinician would be cautious in using the SSRS-SSF to measure change in empathy skills in another Year 7 Australian student, due to low confidence that empathy scores on reassessment reflect baseline scores [19]. Because Pearson’s (r) and ICC are expressed in scale format and not the same units of measurement as the tool [20], they have limited clinical applicability beyond highlighting the psychometric rigor with which a tool measures test–retest scores.

Staying with the above example, if one looks closely at the SD of the empathy and assertion subscales, we note that girls’ empathy scores were less spread around the mean (M_*1*_= 16.66, SD_1_= 1.93; M_*2*_= 16.27, SD_*2*_= 2.04), when compared to boys’ assertion scores (M_*1*_= 13.24, SD_*1*_= 3.11; M_*2*_= 13.90, SD_*2*_= 3.10). So, as a group, girls’ scored more homogeneously, than boys did on assertion behaviours (more heterogeneous). The wider spread of boys’ scores on the assertion subscale resulted in the magnitude of Pearson’s (r) and ICC being greater [2,12,21]. Even a high value does not provide the surety of test–retest scores being interchangeable. Practitioners should therefore be extremely judicious in selecting an outcome tool based on the reported test–retest scores as they could be misleading. Additionally, Pearson’s (r) and ICC do not quantify the unaccounted variation in scores in the measurement scale of the outcome measure (i.e., they do not explain the unaccounted 47% of empathy scores in the measurement units of the empathy subscale), so the clinical interpretation of their score is limited.

We computed the coefficient of repeatability (CR) or the Smallest Real Difference (SRD) to index the measurement error or the smallest possible change in subscale and total social skills scale scores that represents true/real change [2,10,11,17]. The CR accounts for both random and systematic error in its scores. In the above example, the CR of the empathy subscale for girls was therefore smaller (± 3.81) than that for boys on the assertion subscale (± 4.21). Based on the CR, a practitioner using the SSRS-SSF empathy subscale with a year 7 girl in Australia would need to see a change of at least 3.81 units at re-assessment to be 95% confident that the girl had, in fact, benefited from the intervention. A change of less than 3.81 might simply be due to the inherent mechanical inaccuracy of the empathy subscale, which is unable to reliably detect change of less than 3.81 units. The abovementioned example demonstrates the advantage of considering ME as computed by the CR over Pearson’s (r) or ICC.

The statistically significant difference in mean score as a marker of change (as measured by the *t-*statistic), needs to be interpreted in the light of the tool’s rather larger ME. Based on the *t-*statistic presented in Table 1, boys were found to be more assertive on retest (Mean bias = 0.66; *p* = 0.005) [22]. The interpretation is that there was evidence of an increase in this scale from baseline to the 4-week retest, and this increase was statistically significantly different from zero. The CR (±4.21) means that any individual boy is expected to give readings on this scale which are within 4.21 units of this bias. The bias would always need to be interpreted in terms of its clinical significance, and it may be that a difference of this amount, being small relative to the range of values expected for the individual, is of limited interest [17]. In terms of an implication, the statement of the tool’s ME assists the researcher who wishes to develop some intervention addressing the tool’s outcome. The tool’s ME is essential in order to calculate the sample size required to demonstrate an effect of an intervention that is intended to reach some clinically relevant threshold.

To date, there exists no consensus on what the acceptable value of a correlation coefficient ought to be to inform tool selection [4,12]. Tool developers often cite Shrout and Fleiss study on reliability to support claims that a clinically acceptable correlation is 0.75 or 0.80 or greater [13]. Shrout and Fleiss’ categorisation is critiqued in the sports sciences and medicine because they did not assess the utility of the recommended correlations [4]. It has been suggested that as a general rule, a value of over 0.90 should be considered high, between 0.80 and 0.90 as moderate, 0.80 and below insufficient while using an instrument for individual decision-making [19]. Whilst such a conservative stance has been adopted in sports and medical sciences, it seems that sociological and behavioural scientists use lower relative reliability thresholds [1]. As highlighted by the illustrations in this paper, even a high correlation value between test–retest scores could be misleading. Best practice guidelines in measurement literature recommend use of both relative and absolute reliability ME indices [2,10,12].

Unlike relative reliability indices, to date there is no formulaic approach to benchmark ME. This means that there exists no statistical method to decide whether a ME of ± 4.21 in relation to the range of scores on the assertion subscale (assertion subscale range = 0 - 20 units) is wide or small. Thus, although ME sets the boundaries of the minimal delectable true change of an outcome measure, it holds limited clinical importance beyond that function.

ME helps clinicians decide on a best practice level whether the observed change in a client’s performance is true [11]. The value of the ME does not provide insight into the critical clinical question of “How large should change in an outcome be, to be deemed clinically important (i.e., to have an impact on patient care)?” The latter is determined by a statistic called the Minimum Clinically Important Difference of a tool (MCID). The MCID is related to responsiveness or the ability of a tool to detect clinically relevant changes over time and is decided on clinical grounds (and not based on statistical analysis) [23]. An outcome measurement that shows high ME (i.e., variability within stable subjects) would be considered to have poor responsiveness. In that regard, reproducibility (test–retest reliability) is a necessary condition of responsiveness.

It is vital that the ME of an outcome tool be corroborated against its MCID before clinicians decide to use the tool to measure change in an intervention study. For example, Schuling et al. [24], reported no changes in sickness impact profile scores (SIP) during the first 6-months post-stroke; but during the same period the Barthel Activities of Daily Living scores changed significantly. The authors concluded that the SIP was not responsive (sensitive) enough to detect modest improvement in a consecutive cohort of acute stroke patients [24]. In practice, if the ME of an outcome measure is wider than its MCID (i.e., CR > MCID), it is likely that the outcome measure will mask functional change [2,11] and inaccurately report that there is no true change in outcome when in fact the intervention had been effective (i.e., the occurrence of a Type II error or false negative). 
